# Development and validation of a predictive model for left ventricular diastolic improvement following catheter ablation in atrial fibrillation with diastolic dysfunction: a retrospective analysis

**DOI:** 10.3389/fcvm.2025.1689041

**Published:** 2026-01-12

**Authors:** Ying Cao, Zexi Li, Aobo Gong, Fanghui Li, Xianjin Hu, Bangjiaxin Ren, Wenjie Li, Rui Zeng

**Affiliations:** Department of Cardiology, West China Hospital, Sichuan University, Chengdu, China

**Keywords:** atrial fibrillation, catheter ablation, cross-validation, echocardiography, left ventricular diastolic dysfunction, predictive model

## Abstract

**Background:**

The impact of catheter ablation on left ventricular diastolic function in patients with atrial fibrillation (AF) and left ventricular diastolic dysfunction (LVDD) remains poorly characterised. There are no robust tools available to predict improvement in LVDD post-ablation.

**Methods:**

The retrospective study enrolled 141 patients diagnosed with AF and LVDD undergoing ablation. LVDD improvement was defined as a reduction of at least one grade in LVDD grade (LVDDG) within 12 ± 3 months after ablation. The predictive model was constructed using a logistic regression with 10-fold validation with stratification. The performance of the model was evaluated using ROC analysis (AUC), calibration plots, and decision curve analysis (DCA). An exploratory sensitivity analysis applying the updated 2025 ASE recommendations for LVDD assessment was additionally performed.

**Results:**

49.6% (70/141) of patients showed improvement in LVDD. The predictive model incorporated four independent predictors: higher baseline LVDDG, lower left ventricular mass index (LVMI), lower left ventricular ejection fraction (LVEF), and absence of a history of stroke. The AUC was 0.786 (95% CI 0.760–0.809) in the training cohort and 0.756 (95% CI: 0.677–0.835) in the validation cohort, with acceptable calibration and net clinical benefit across a restricted range of threshold probabilities. In the sensitivity analysis using the redefined LVDD endpoint, discrimination remained comparable (AUC 0.783, 95% CI 0.703–0.856).

**Conclusions:**

A novel model integrating LVDDG, LVMI, LVEF and stroke history effectively predicts post-ablation LVDD improvement in AF patients with LVDD, aiding patient selection for ablation and guiding adjunctive therapies.

## Introduction

Atrial fibrillation (AF) can coexist with left ventricular diastolic dysfunction (LVDD) (1, 2), which can progress to symptomatic heart failure, adversely affecting quality of life and survival (3, 4).Catheter ablation is a cornerstone intervention for symptomatic AF (5, 6). However, the impact of ablation on LVDD in patients presenting with both conditions remains inadequately characterized. While preliminary evidence from a limited number of studies suggests that some patients experience improvement in left ventricular diastolic function following the procedure (7, 8), the factors determining this response in the AF population with LVDD remain poorly understood. The potential for LVDD improvement is of clinical significance, as it may contribute to a reduction in major adverse cardiovascular events, including acute HF exacerbations/hospitalizations and stroke (9, 10). Numerous factors are known to influence left ventricular diastolic function, such as diabetes mellitus (DM), coronary artery disease (CAD), chronic kidney disease, stroke and age (11–14). However, it is unclear what factors can reliably predict changes in LVDD following AF ablation in patients with pre-existing LVDD. Robust predictive models identifying patients who tend to gain this specific benefit, beyond the restoration of sinus rhythm, are notably lacking.

Therefore, we aimed to develop and validate a preliminary predictive model using easily accessible clinical parameters to identify AF patients with LVDD who are more likely to benefit from catheter ablation in terms of diastolic improvement, which may inform patients counseling and guide future research.

## Methods

### Patient population

This retrospective study consecutively screened patients who underwent catheter ablation for AF at West China Hospital of Sichuan University between June 2018 and October 2022. Inclusion criteria comprised: age ≥18 years; (2) diagnosis of AF; (3) undergoing catheter ablation for AF at West China Hospital; and (4) the presence of LVDD documented by pre-procedural transthoracic echocardiography (TTE) performed within 7 days prior to the procedure. Exclusion criteria were: (1) absence of a comprehensive follow-up TTE(including M-mode, two-dimensional, and Doppler assessments) performed within the 1-year window (defined as 12 ± 3 months post-procedure); (2) Pre-procedural or 1-year post-procedural TTE not performed during sinus rhythm; and (3) incomplete baseline clinical data.

### Evaluation of LVDD

LVDD was defined according to current guidelines and prior evidence (15, 16), as meeting ≥2 of the following criteria: Septal E/e’ ratio >15, septal e’ velocity <7 cm/s, maximal tricuspid regurgitation velocity (TRV_max_) > 2.8 m/s, left atrial volume index (LAVi) > 34 mL/m² or left atrial diameter (LAD) > 34 mm. To further evaluate the severity of LVDD, a three-stage grading system was applied based on comprehensive echocardiographic parameters: LVDD Grade 1 (LVDDG 1): E/A ratio ≤0.8 + E-wave velocity ≤50 cm/s or 0.8 < E/A ratio <2 with ≤ 1 abnormal parameter among TRV_max_ >2.8 m/s, septal E/e’ > 15, LAVi >34 mL/m² or LAD >34 mm; LVDD Grade 2 (LVDDG 2): 0.8 < E/A ratio <2 with ≥2 abnormal parameters from the above list; LVDD Grade 3 (LVDDG 3): E/A ratio ≥2.

Improvement in LVDD was defined as a reduction of at least one grade in LVDDG when comparing postoperative to preoperative assessments.

### Data collection

Baseline data were retrospectively collected from patients with AF and concomitant LVDD undergoing catheter ablation. The following variables were included in the preliminary screening list: Demographic characteristics: gender, age, smoking history and alcohol consumption history; clinical characteristics: AF type (paroxysmal/persistent), comorbidities (hypertension, DM, CAD) and history of stroke and prior history of AF ablation; echocardiographic parameters: LVDDG, left ventricular ejection fraction (LVEF), left ventricular mass index (LVMI), relative wall thickness (RWT), mitral regurgitation (MR) severity grade and tricuspid regurgitation (TR) severity grade; serum biomarkers: estimated glomerular filtration rate (eGFR).

### Ablation procedure

All procedures were performed under local anesthesia for radiofrequency and cryoablation cases. Pulmonary vein isolation (PVI) was achieved in each patient. The following adjunctive ablation strategies were performed in certain patients at the operator's discretion: posterior wall box isolation, left atrial roof line ablation, mitral isthmus line ablation, tricuspid isthmus line ablation, superior vena cava (SVC) segmental ablation, ablation of complex fractionated atrial electrograms (CFAEs) and coronary sinus ostial ablation. Left atrial appendage closure was implemented when deemed necessary for thromboembolic risk reduction.

### Follow-up

At the 1-year follow-up interval, patients in the study underwent comprehensive assessment evaluating two endpoints: (1) Improvement in left ventricular diastolic function, determined through comparative assessment of LVDDG at pre- and post-procedure using standardized transthoracic echocardiography incorporating M-mode, 2-dimensional imaging and Doppler parameters. (2) Ablation efficacy assessed by monitoring atrial fibrillation recurrence status throughout the 1-year period via 12-lead electrocardiograms and 24- or 72-hour ambulatory electrocardiographic (Holter) records, with recurrence defined according to guideline recommendations as any documented episode of atrial fibrillation, atrial flutter or atrial tachycardia lasting ≥30 s occurring after the 3-month blanking period post-ablation (17).

### Statistical methodology

In view of the moderate sample size, the dataset was partitioned into training and validation sets using 10-fold random splitting. Stratification was performed by outcome status to ensure that the proportion of characteristics was maintained consistently across all folds, thus preventing sampling bias. For full reproducibility of the data partitioning and analysis, a fixed random seed [set.seed(123)] was used for the split, and baseline balance in the representative fold was evaluated using standardized mean differences (SMDs).

Baseline characteristics between outcome-stratified subgroups were compared using independent samples *t*-tests or Mann–Whitney *U*-tests for continuous variables (depending on distribution normality), and chi-square or Fisher's exact tests for categorical variables as appropriate.

The selection of variables were performed using stepwise logistic regression analysis guided by the Bayesian Information Criterion (BIC). A nomogram was constructed to visualize predictor contributions. Receiver operating characteristic (ROC) curve analysis with area under the curve (AUC) calculation (95% CI), sensitivity and specificity for discrimination evaluation of the model. Calibration plots comparing predicted probabilities against observed outcomes. Decision curve analysis (DCA) quantifying net benefit across threshold probabilities for clinical utility assessment of the model. Statistical significance was defined as two-sided *P* < 0.05. Additionally, a exploratory sensitivity analysis was performed in which baseline and follow-up LVDDG, and outcome status, were rederived using an updated ASE recommendations (18), permitted by the available echocardiographic data in the absence of left atrial strain measurements. A corresponding nomogram and ROC curve with AUC (95% CI estimated from 1,000-bootstrap resamples) were also conducted. All analyses were performed using R software (version 4.3.2).

## Results

### Baseline charateristics

Following rigorous application of inclusion and exclusion criteria, 141 patients with AF and concomitant LVDD undergoing catheter ablation were ultimately enrolled. Baseline clinical, echocardiographic, procedural and pharmacological characteristics for the overall cohort and outcome-stratified subgroups are presented in Table 1. A total of 17 variables were screened as potential predictors of post-procedural LV diastolic function improvement: gender, age, smoking history, alcohol consumption history, AF type, hypertension, DM, CAD, history of stroke, prior AF ablation history, LVDDG, LVEF, LVMI, RWT, MR severity grade, TR severity grade, and eGFR. Because LVDDG is a three-category variable, its inclusion as a categorical predictor in the model increased the total variable count by one beyond the original 17 potential predictors (18 in total).

**Table 1:** Baseline characteristics of the study population.

Characteristic	Overall (*n* = 141)	Negative group (*n* = 71)	Positive group (*n* = 70)	*p*-value
Age (years), median [IQR]	68.00 [60.00, 75.00]	70.00 [64.50, 76.00]	67.00 [59.00, 72.00]	0.075
LVMI (g/m²), median [IQR]	102.99 [86.04, 121.47]	110.83 [88.15, 126.47]	95.84 [85.02, 114.06]	0.021
RWT, median [IQR]	0.38 [0.35, 0.42]	0.40 [0.36, 0.43]	0.37 [0.34, 0.40]	0.005
LVEF (%), median [IQR]	66.00 [62.00, 70.00]	67.00 [62.00, 71.50]	65.00 [61.00, 69.00]	0.273
eGFR (mL/min/1.73 m²), median [IQR]	79.64 [65.33, 90.86]	77.91 [63.69, 90.94]	80.86 [68.52, 90.67]	0.415
Gender, *n* (%)				1.000
Male	70 (49.6)	35 (49.3)	35 (50.0)	
Female	71 (50.4)	36 (50.7)	35 (50.0)	
Atrial fibrillation type, *n* (%)				0.645
Paroxysmal	122 (86.5)	60 (84.5)	62 (88.6)	
Persistent	19 (13.5)	11 (15.5)	8 (11.4)	
LVDDG, *n* (%)				<0.001
1	65 (46.1)	43 (60.6)	22 (31.4)	
2	56 (39.7)	26 (36.6)	30 (42.9)	
3	20 (14.2)	2 (2.8)	18 (25.7)	
Hypertension, *n* (%)				1.000
No	65 (46.1)	33 (46.5)	32 (45.7)	
Yes	76 (53.9)	38 (53.5)	38 (54.3)	
Diabetes mellitus, *n* (%)				0.126
No	117 (83.0)	55 (77.5)	62 (88.6)	
Yes	24 (17.0)	16 (22.5)	8 (11.4)	
Coronary artery disease, *n* (%)				0.383
No	127 (90.1)	66 (93.0)	61 (87.1)	
Yes	14 (9.9)	5 (7.0)	9 (12.9)	
History of stroke, *n* (%)				0.194
No	125 (88.7)	60 (84.5)	65 (92.9)	
Yes	16 (11.3)	11 (15.5)	5 (7.1)	
Smoking, *n* (%)				0.415
No	114 (80.9)	55 (77.5)	59 (84.3)	
Yes	27 (19.1)	16 (22.5)	11 (15.7)	
Drinking, *n* (%)				0.261
No	119 (84.4)	57 (80.3)	62 (88.6)	
Yes	22 (15.6)	14 (19.7)	8 (11.4)	
Mitral regurgitation, *n* (%)				1.000
No	103 (73.0)	52 (73.2)	51 (72.9)	
Yes	38 (27.0)	19 (26.8)	19 (27.1)	
Tricuspid regurgitation, *n* (%)				0.442
No	92 (65.2)	49 (69.0)	43 (61.4)	
Yes	49 (34.8)	22 (31.0)	27 (38.6)	
Redo ablation, *n* (%)				0.645
No	122 (86.5)	60 (84.5)	62 (88.6)	
Yes	19 (13.5)	11 (15.5)	8 (11.4)	
Ablation energy modality, *n* (%)				0.772
Radiofrequency ablation	130 (92.2)	65 (91.5)	65 (92.9)	
Cryoablation	11 (7.8)	6 (8.5)	5 (7.1)	
LAAO, *n* (%)				0.869
No	112 (79.4)	56 (78.9)	56 (80.0)	
Yes	29 (20.6)	15 (21.1)	14 (20.0)	
Additional ablation, *n* (%)				0.973
No	121 (85.8)	61 (85.9)	60 (85.7)	
Yes	20 (14.2)	10 (14.1)	10 (14.3)	
ACEI/ARBs, *n* (%)				0.652
No	86 (61.0)	42 (59.2)	44 (62.9)	
Yes	55 (39.0)	29 (40.8)	26 (37.1)	
Beta - blockers, *n* (%)				0.539
No	58 (41.1)	31 (43.7)	27 (38.6)	
Yes	83 (58.9)	40 (56.3)	43 (61.4)	
SGLT2 inhibitors, *n* (%)				0.496
No	139 (98.6)	69 (97.2)	70 (100.0)	
Yes	2 (1.4)	2 (2.8)	0 (0.0)	
MRAs, *n* (%)				1.000
No	131 (92.9)	66 (93.0)	65 (92.9)	
Yes	10 (7.1)	5 (7.0)	5 (7.1)	
Diuretics, *n* (%)				0.563
No	129 (91.5)	64 (90.1)	23(32.9)	
Yes	12 (8.5)	7 (9.9)	5 (7.1)	

Data are presented as *n* (%) for categorical variables or median [IQR] for continuous variables. p-values were calculated using the Chi-square test, Fisher's exact test, or Wilcoxon rank-sum test as appropriate. IQR, interquartile range; LVDD, left ventricular diastolic dysfunction; LVMI, left ventricular mass index; RWT, relative wall thickness; LVEF, left ventricular ejection fraction; eGFR, estimated glomerular filtration rate; LAAO, transcatheter left atrial appendage occlusio; ACEI, angiotensin-converting enzyme inhibitor; ARB, angiotensin receptor blocker; MRA, mineralocorticoid receptor antagonist; SGLT2, sodium–glucose cotransporter-2.

We implemented stratified 10-fold cross-validation to assess model performance. In each iteration, the dataset was partitioned into 90% training and 10% validation subsets, with stratification by outcome status. A fixed random seed (123) guaranteed reproducibility. We examined baseline characteristics of the training and validation subsets in a representative fold (Fold 5) of the stratified 10-fold cross-validation. As shown in Supplementary Table S2, most variables had standardized mean differences (SMDs) in the small-to-moderate range (approximately 0.1–0.3), whereas an SMD of 0.664 was observed for tricuspid regurgitation, likely reflecting random variation in the very small validation subset (*n* = 14) rather than systematic distortion.

### Variable selection

To more stably identify variables predictive of LVDD improvement following AF ablation, stepwise logistic regression was employed, guided by the Bayesian Information Criterion (BIC) within the training cohort. This procedure was iterated across ten training sets generated through 10-fold random splitting. In accordance with this approach, the variables that were repeatedly selected (appearing in >1 model across the ten iterations) were retained for the subsequent development of predictive model. The key predictors that were identified in this study included LVDDG 2/3 (selected in all 10 models), LVMI (selected in 9 models), History of Stroke (selected in 6 models) and LVEF (selected in 4 models).

### Logistic regression model development and validation

**Figure 1:**
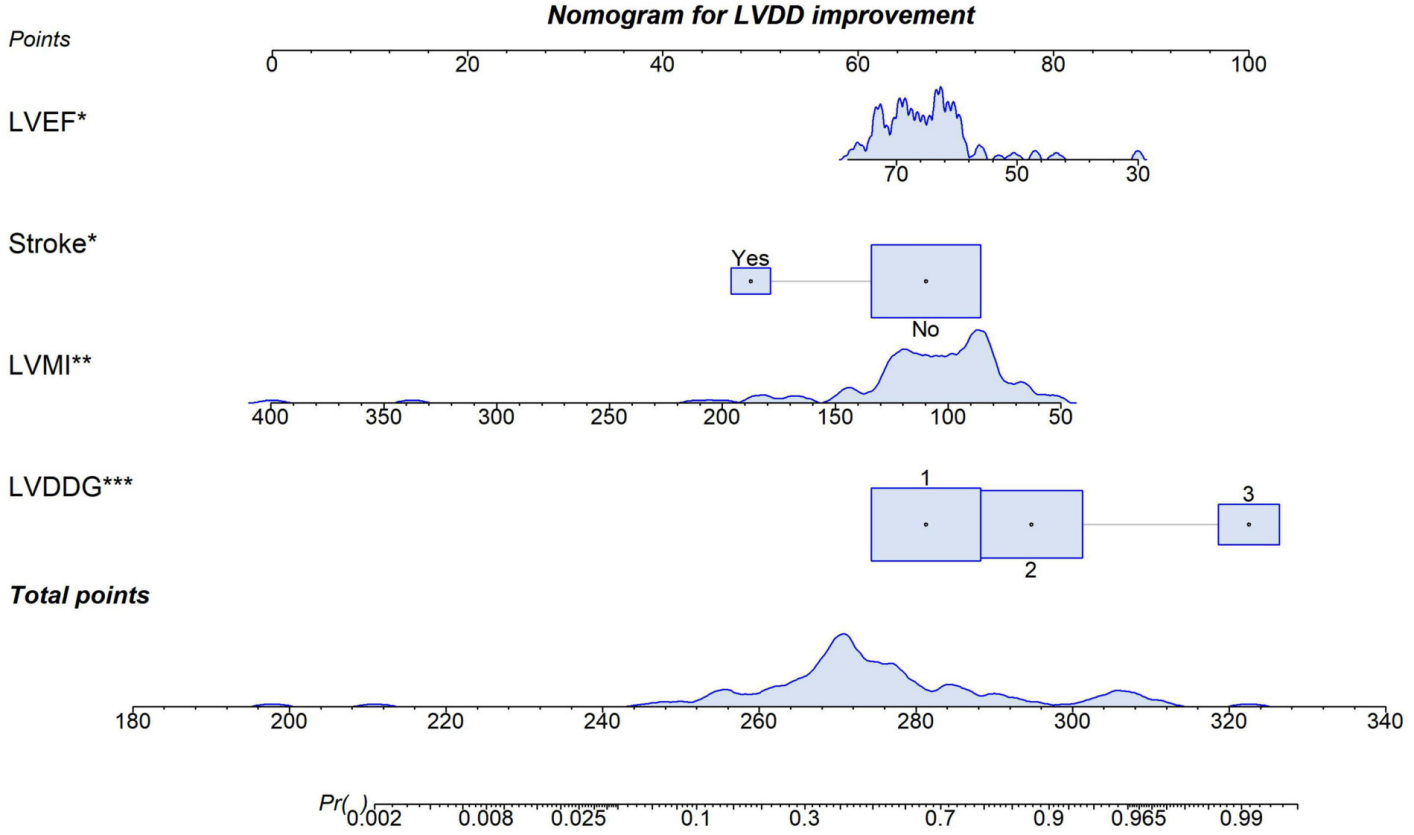
Predictive nomogram for improvement in LVDD after AF ablation. *p < 0.05 for a variable in the model.**p < 0.05. ***p < 0.005.

Multivariable logistic regression analysis incorporating the key predictors (LVDDG, LVMI, history of stroke and LVEF) demonstrated statistically significant associations with improvement in LVDD following ablation (Table 2). These factors, identified as independent predictors of post-ablation LVDD improvement, underscore critical clinical determinants of diastolic recovery. Notably, patients exhibiting a higher baseline LVDDG manifested a substantially increased likelihood of achieving post-procedural LVDD improvement. A point-based clinical nomogram was then constructed from this model (Figure 1). For each patient, values of LVDDG, LVMI, LVEF and stroke history are converted into points, summed to a total score and mapped to the bottom axis to yield the predicted probability of LVDD improvement.

**Table 2:** Binary logistic regression analysis for baseline characteristics.

Variable	B	SE	Z	OR	95% CI	*p*-value
LVDDG (level 2 vs. 1)	1.054	0.417	2.53	2.869	1.287–6.653	0.012
LVDDG (level 3 vs. 1)	3.226	0.892	3.62	25.183	5.414–200.518	<0.001
History of Stroke	−1.752	0.716	−2.45	0.173	0.037–0.647	0.014
LVMI	−0.023	0.008	−2.97	0.978	0.962–0.991	0.003
LVEF	−0.060	0.028	−2.13	0.941	0.886–0.991	0.033

OR, odds ratio; CI, confidence interval.

**Figure 2:**
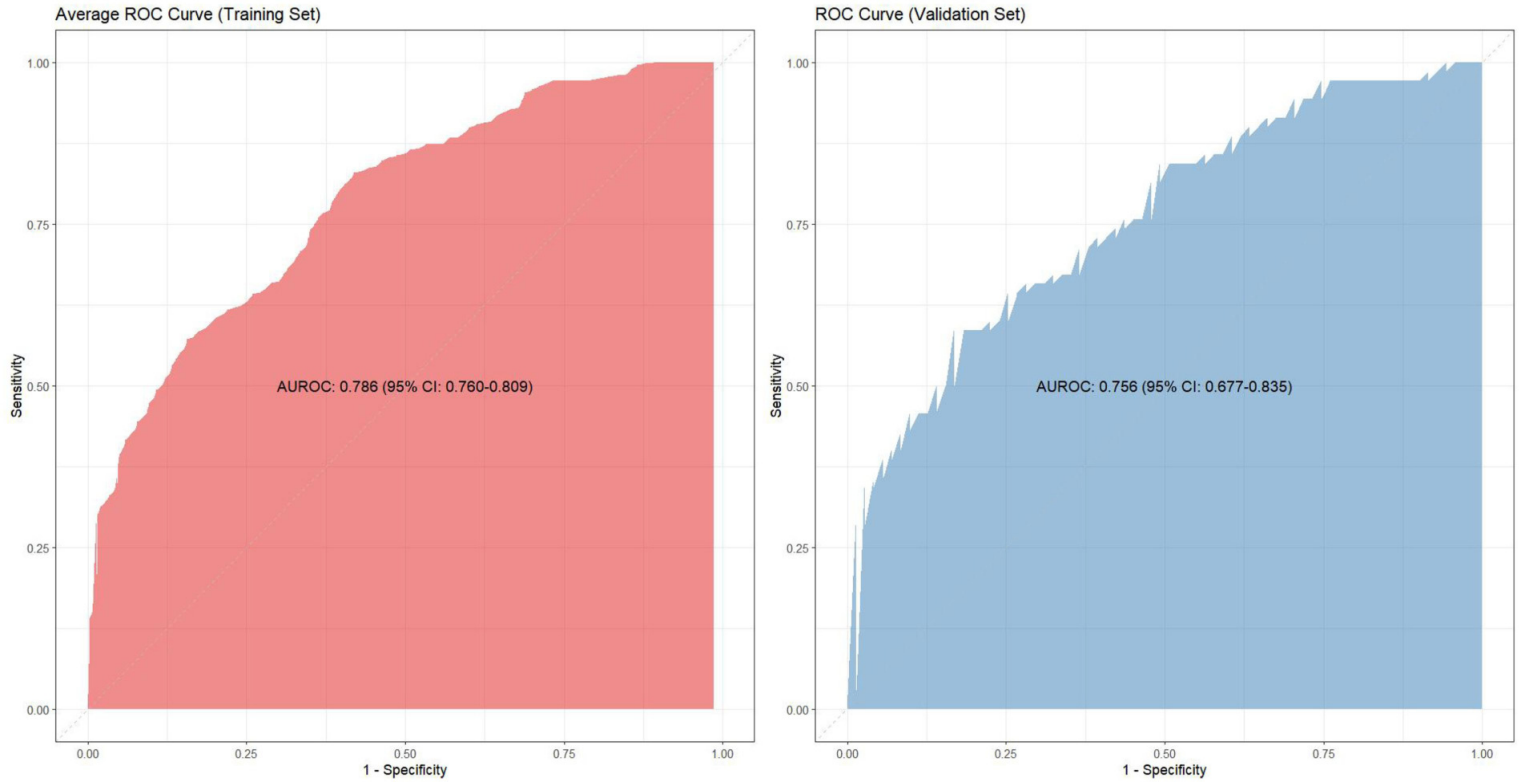
The ROC curves of training cohort and validation cohort.

Given the substantial overlap among the ten training subsets (each comprising 90% of the total data), the average receiver operating characteristic (ROC) curve was generated by computing mean specificity and sensitivity values across all cross-validation folds. This yielded a mean area under the curve (AUC) of 0.786 (95% CI 0.760–0.809). In contrast, the mutually independent validation subsets facilitated robust performance estimation through integration of predicted outcomes across all ten validation iterations, achieving a validation AUC of 0.756 (95% CI 0.677–0.835) (Figure 2). The model demonstrated high specificity (83.1%) but moderate sensitivity (58.6%) in the validation cohort.

**Figure 3:**
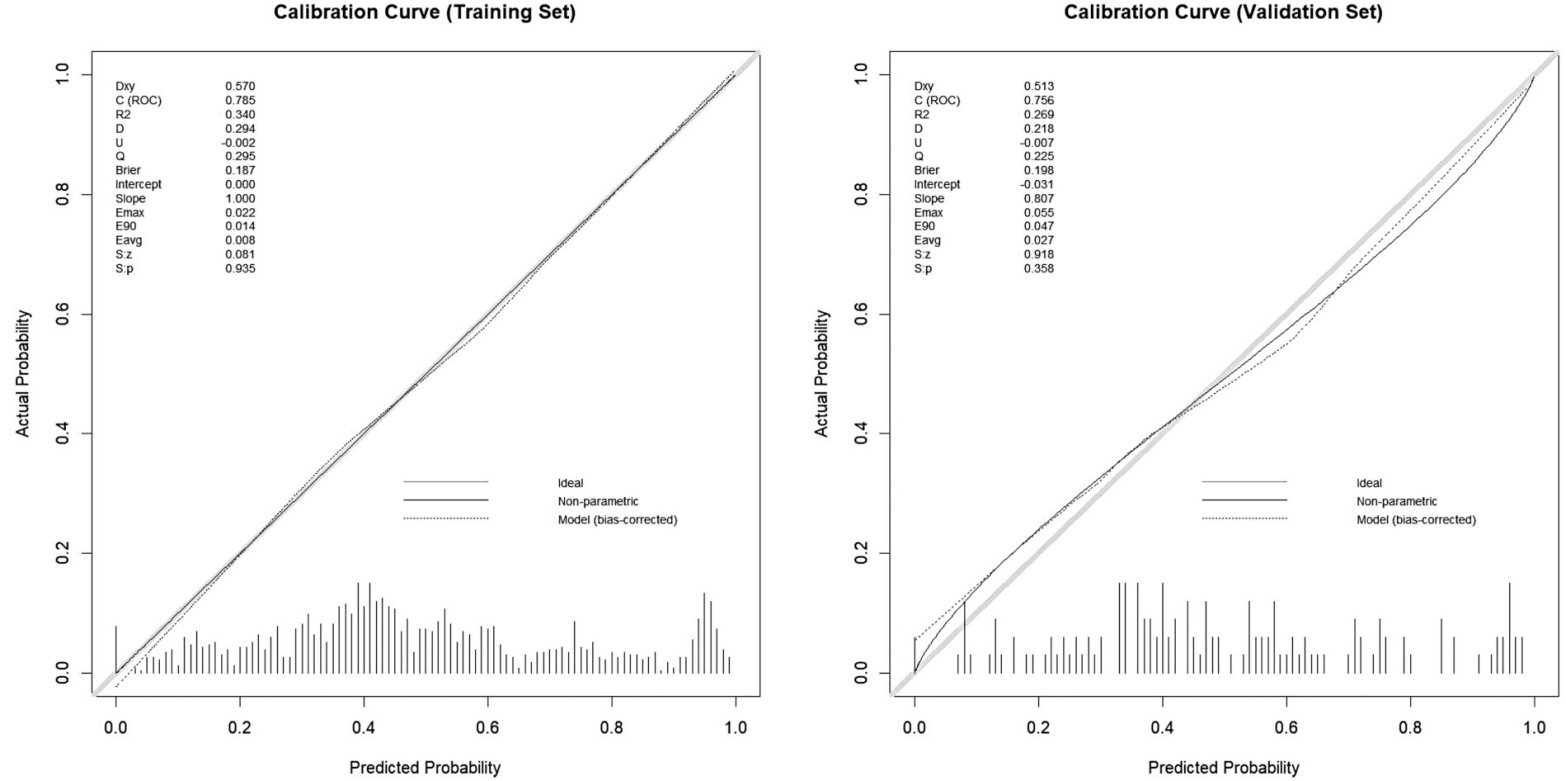
The calibration curves of training cohort and validation cohort.

**Figure 4:**
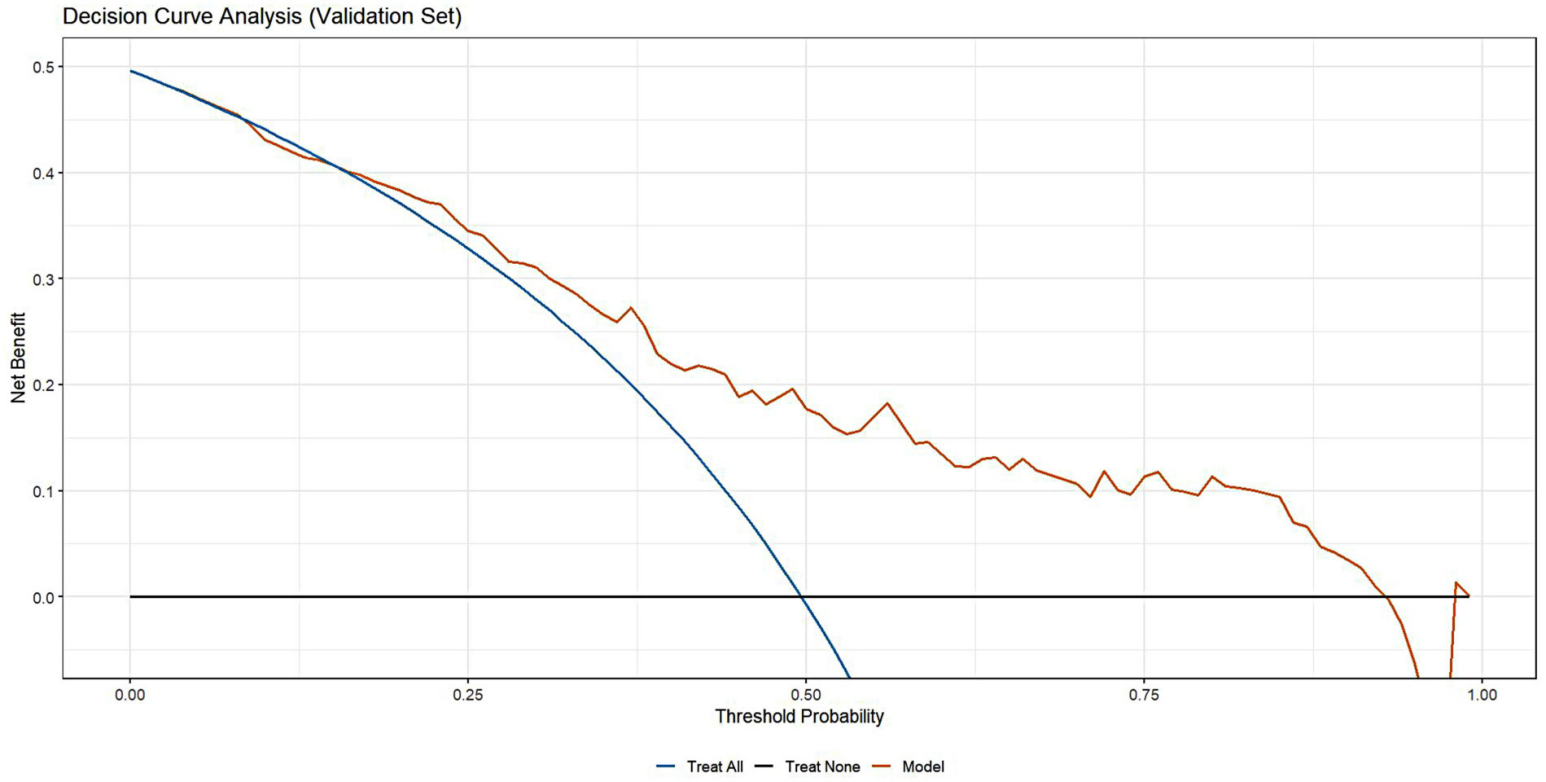
The clinical decision curve of validation cohort.

The model demonstrated acceptable accuracy overall. The Mean Absolute Error (MAE) and Brier score served as key metrics for evaluating model performance (Figure 3). Analysis revealed a discrepancy of 0.11 in MAE between two calibration lines. The Brier score, measuring calibration accuracy, was 0.187 in the training set and 0.198 in the validation set. The model successfully passed the overall goodness-of-fit test (*χ*², *p* = 0.358). Furthermore, the average predicted risk in the validation cohort (49.96%) closely aligned with the observed event rate (49.65%), demonstrating a minimal discrepancy of 0.31%. Based on the optimal cutoff derived from the Youden index, the estimated PPV was ∼77% and the NPV was ∼67%. However, a validation cohort slope of 0.807 was observed, suggesting some degree of overfitting. Specifically, good agreement between predicted and observed risk was observed mainly in the mid-range of predicted probabilities (∼0.40–0.55). Consistently, DCA (Figure 4) indicated that the model provided distinguished net benefit chiefly for threshold probabilities between about 0.20 and 0.50.

### Impact of procedure-related characteristics on LVDD improvement after ablation

To determine whether procedure-related characteristics influenced LVDD improvement following AF ablation, procedural variables[ablation energy modality, transcatheter left atrial appendage occlusion (LAAO), and additional non-PV ablation] were incorporated into a multivariable logistic regression analysis alongside the predictors from our previously established predictive model (LVDDG, History of Stroke, LVMI, LVEF). The results (Table 3) demonstrated no significant association between these procedural characteristics and post-ablation LVDD improvement (all *P* > 0.05).

**Table 3:** Multivariable analysis including procedure-related variables.

Variable	OR	95% CI	*p*-value
History of stroke	0.17	0.04, 0.63	0.012
LVDDG (level 2 vs. 1)	2.95	1.30, 6.96	0.011
LVDDG (level 3 vs. 1)	26.8	5.69, 216	<0.001
LVEF	0.94	0.89, 0.99	0.041
LVMI	0.98	0.96, 0.99	0.003
LAAO	0.86	0.31, 2.33	0.759
Ablation energy modality	1.46	0.31, 7.14	0.606
Additional ablation	1.36	0.42, 4.38	0.620

### Sinus rhythm restoration after ablation

Electrocardiogram-documented atrial fibrillation recurrence status at 1-year post-ablation could not be ascertained for 5 patients despite comprehensive review of inpatient/outpatient records and telephone follow-up attempts. Among the 136 evaluable patients, 42 experienced AF recurrence, yielding a AF-free rate of 69.1% at one year after ablation.

### Exploratory sensitivity analysis with the updated LVDD definition

In the exploratory sensitivity analysis, rederiving baseline and follow-up LVDDG based on the updated 2025 ASE recommendations partialy altered the distribution of LVDD severity, with most patients classified as grade 2(58.8%) and a small number reclassified as grade 0(2.8%), who were excluded from this analysis. Under the adapted definition, the prevalence of LVDD improvement shifted from approximately 1:1 in the primary analysis to about 1:1.5 (improved vs. non-improved). We applied outcome-specific weights so that the weighted distribution of LVDD improvement approximated a 1:1 ratio and then refitted the multivariable logistic regression model. We found baseline LVDDG and LVMI remained independent predictors. The associations of LVEF (*p* = 0.25) and prior stroke (*p* = 0.09) were attenuated and did not reach statistical significance, although the direction of effect was consistent with the primary model (Supplementary Table S1). A corresponding nomogram illustrating the contribution of LVDDG, LVMI, LVEF and stroke history under the updated LVDD definition (Supplementary Figure S1). Discrimination was comparable to that of the original model, with an AUC of 0.783 and a 95% CI of 0.703–0.856 estimated by 1,000-bootstrap resampling (Supplementary Figure S2).

## Discussion

AF combined with LVDD is significantly associated with an increased risk of adverse cardiovascular events. While catheter ablation serves as an important curative procedure for AF, evidence regarding its impact on left ventricular diastolic function in patients with coexisting LVDD remains limited (19). This study evaluated postoperative improvement in LVDD using a comprehensive index (LVDDG). The results demonstrated that left ventricular diastolic function improved postoperatively in 49.6% of patients with AF and LVDD, providing evidence supporting the potential of catheter ablation to improve diastolic function in this patient population.

We successfully developed and rigorously validated a novel model for predicting improvement in LVDD following AF ablation, and constructed a nomogram visualizing the weight of each variable. LVDDG, LVMI, LVEF and history of stroke were all identified as independent predictors of post-procedural diastolic function improvement. A higher LVDDG was found to be a strong independent positive predictor of LVDD improvement post-ablation. Conversely, higher LVMI, higher LVEF, and history of stroke were independent negative predictors of LVDD improvement.

### Key predictors and mechanistic interpretation

LVDDG was a powerful predictor of LVDD improvement after AF ablation. In this study, the majority of patients with higher LVDDG were AF patients exhibiting E/e’ > 15. This finding shows a degree of consistency with the results of Kim et al. (20), which reported that E/e’ ≥ 15 was an independent predictor of E/e’ improvement at 1 year after ablation. Furthermore, our results suggest that patients with most severe LVDD (LVDDG 3), those with a restrictive filling pattern, are more likely to experience improvement in diastolic function following ablation. We note that several studies have reported a reduction in E-wave velocity post-ablation (20, 21), supporting a decrease in the prevalence of E/A ≥ 2 post-procedure. This provides some basis for the potential conversion of a restrictive filling pattern to less severe diastolic dysfunction or even normal function. Beyond diastolic function parameters, LVEF was also independently associated with diastolic improvement. Analysis indicated that a reduced LVEF predicted a greater likelihood of diastolic function improvement post-ablation. Kucukdurmaz et al. (22) demonstrated that E/e’ remained unchanged in the normal LVEF group (*n* = 24; pre- vs. post-12 months: 9 ± 3 vs. 9 ± 4), while it decreased from 13 ± 7 pre-procedure to 11 ± 4 at 12 months post-procedure in the low LVEF group (*n* = 11). Although it was reported the improvement in the low LVEF group was not statistically significant, it was likely related to the small subgroup size. Thus, this result provides support for the positive predictive role of reduced LVEF on LVDD improvement. Parameters related to left ventricular structure were also incorporated into the model to aid prediction. We found that higher LVMI was a negative predictors of LVDD improvement after ablation. Elevated LVMI signifies left ventricular hypertrophy and possible cardiomyopathy. Gottlieb et al. (23) indicated an increased risk of heart failure hospitalization post-ablation in patients with hypertrophic cardiomyopathy, supporting the notion that increased LVMI is unfavorable for post-procedural LVDD improvement. History of stroke was also an independent negative predictor of LVDD improvement after ablation in this study. Direct evidence regarding post-ablation changes in left ventricular diastolic function specifically in stroke patients remains scarce. Prior researches (12, 24) reported that ischemic stroke negatively impacts left ventricular diastolic function. Our results suggest that the negative effect may persist after ablation in AF patients with a history of ischemic stroke.

Potential underlying mechanisms are analyzed for the four key variables how to influence LVDD improvement following ablation. A higher baseline LVDDG predicted a significantly increased probability of left ventricular diastolic function improvement. The potential mechanism may lie in the significantly reduced left ventricular compliance and heightened dependence on atrial contractile function under conditions of severe diastolic dysfunction. AF itself exacerbates diastolic dysfunction through two pathways: loss of effective atrial contraction, reducing late diastolic active ventricular filling volume; and shortening of diastole due to irregular ventricular rates, further limiting filling time. Following catheter ablation, which reduces AF burden or successfully maintains sinus rhythm, restoration of atrial transport function (25) increases ventricular filling volume. This is particularly crucial for hemodynamic compensation in ventricles with low compliance. Concurrently, the regularization of RR intervals significantly prolongs diastole, creating a time window for slow filling. These combined effects likely contribute to greater reductions in the E/A ratio and improvements in E/e’, particularly pronounced in patients with the poorest compliance (LVDDG 3). Regarding the counterintuitive finding that a reduced LVEF predicted postoperative LVDD improvement, a potential mechanism may be the lack of a significant association between myocardial fibrosis (a factor detrimental to LVDD improvement) and LVEF (26), which also holds consistent with patients with hypertrophic cardiomyopathy (27), alleviating concerns that low LVEF inherently impedes LVDD improvement to some extent. Specifically, diastolic improvement in patients with low LVEF may benefit from greater reserve for systolic function improvement. The restoration of atrial contraction post-ablation enhances preload reserve, utilizing the Frank-Starling mechanism to increase stroke volume and reduce end-systolic volume, thereby diminishing diastolic wall stress. Furthermore, the reduction in AF burden or restoration/maintenance of sinus rhythm post-ablation may improve energy metabolism, enhancing the phosphocreatine-to-ATP ratio, which promotes accelerated myosin ATPase hydrolysis and active relaxation. In this study, a higher LVMI was a negative predictor of LVDD improvement post-ablation. Elevated LVMI typically reflects more severe myocardial hypertrophy and interstitial fibrosis. Although AF ablation may reduce certain cardiac loads, significant hypertrophy and fibrosis are likely less reversible, thereby limiting the degree of LVDD improvement. Notably, RWT, an index often combined with LVMI to assess left ventricular geometric hypertrophy patterns, showed no significant association with post-ablation LVDD improvement in our study. This further suggests the potential reversibility of isolated concentric remodeling (a subtype of left ventricular remodeling characterized by normal LVMI but elevated RWT) following ablation. Thus myocardial hypertrophy and the intrinsic burden of myocardial fibrosis appear to be the core limiting factors for LVDD improvement. A history of stroke was also identified as a negative predictor of LVDD improvement. Stroke contributes to various cardiovascular complications, including heart failure, through autonomic dysregulation, inflammatory activation and myocardial fibrosis (28, 29). This adverse impact may partially counteract the positive changes in left ventricular diastolic function achieved by ablation. Additionally, stroke may lead to reduced patient mobility, thereby increasing cardiac load and promoting remodeling, resulting it difficult to reverse LVDD after ablation.

### Exploratory sensitivity analysis under updated diastolic-function criteria

Because the present study was initiated before the release of the 2025 ASE recommendations, left atrial strain was not routinely acquired, and the updated algorithm could only be approximated using the available Doppler and left atrial size indices. As a consequence, the rederived LVDDGs tended to collapse toward the intermediate category (LVDDG 2), with relatively few patients classified as either no LVDD or advanced LVDD. This “compression toward the middle” inevitably reduces the sensitivity of the grading scheme to detect true changes in diastolic function over time. Notably, even under these suboptimal conditions and a redefined endpoint, baseline LVDDG and LVMI remained independent predictors and the nomogram preserved good discrimination (AUC 0.783, 95% CI 0.703–0.856), highlighting that the severity of diastolic dysfunction and the burden of LV structural remodelling exert a robust influence on LVDD improvement that is not dependent on a specific generation of guidelines. At the same time, the effect of prior stroke persisted with a borderline *p* value (*p* = 0.09) and a directionally consistent odds ratio, suggesting attenuation of statistical power and probably partial mediation of its impact through LVDDG and LVMI rather than a true absence of prognostic value. Taken together, this exploratory sensitivity analysis indicates that LVDDG and LVMI represent definition-robust core determinants of LVDD recovery across diastolic-function frameworks, while LVEF and stroke history function as important contextual modifiers whose quantitative contribution is more sensitive to the exact grading rules and to sample size.

### Clinical implications

From a clinical perspective, our findings suggest that LVDD improvement after AF ablation is not random but can be estimated using a small set of routinely available variables. The nomogram integrates LVDDG, LVMI, LVEF and history of stroke into an individualized probability of diastolic recovery, which can be calculated at the time of pre-procedural echocardiography. Rather than providing a simple “yes/no” recommendation, the tool positions each patient along a continuum of expected benefit. In patients whose predicted probability of LVDD improvement falls in the higher range, the nomogram can strengthen such case for offering ablation, particularly when guideline-based indications are borderline (e.g., intermediate symptom burden, older age, or multiple comorbidities).

Conversely, patients with a low predicted probability of LVDD improvement (characterized by markedly increased LVMI, and/or a history of stroke), are less likely to experience meaningful diastolic reverse remodelling after ablation. However, given the PPV of ∼77% and NPV of ∼67%, the nomogram is notably more effective for ruling in patients with a high probability of improvement than for confidently excluding benefit. Therefore, for these individuals, the model does not serve as an automatic decisive criterion to deny ablation, but it can change the clinical conversation in several ways. First, it prompts more realistic counselling that rhythm control alone may not substantially improve diastolic function or heart failure risk, and that aggressive optimization of blood pressure control, weight management, neurohormonal blockade and other anti-remodelling strategies remains essential. Second, the strong and independent association between prior stroke and reduced likelihood of LVDD improvement highlights a possible brain–heart axis of adverse remodelling and supports closer multidisciplinary follow-up in this subgroup, where the focus may need to be broadened from rhythm control alone to integrated vascular and cardiac risk management.

## Limitation

Our study has certain limitations. First, this was a single-center and retrospective study, carrying the potential for selection and information bias. Moreover, the limited sample size and the predominance of patients with paroxysmal AF may restrict the generalizability of the findings to some extent. Additionally, our model exhibited a degree of overfitting in the validation cohort. Although rigorous methods, such as cross-validation, were employed during development, future research is needed to validate the model in an independent, larger external cohort to assess its true performance and generalizability.

## Perspectives

Future research should focus on several key areas: First, large-scale, multicenter and prospective studies incorporating external validation are essential. These studies should integrate a broader range of clinical variables, advanced imaging indices and systematically implement the full 2025 ASE diastolic-function protocol into the model to enhance its accuracy and generalizability. Furthermore, future studies could expand the scope of long-term outcome measures by incorporating endpoints such as heart failure hospitalization rates and quality of life assessment scores. Additionally, interventional studies stratified based on the predicted likelihood of LVDD improvement are warranted. For patients with a low probability of LVDD improvement, research should investigate whether adjuvant pharmacologic or non-pharmacologic interventions combined with ablation can ameliorate LVDD and enhance ablation efficacy. For patients with a high probability of LVDD improvement, studies should examine whether early ablation leads to improvements in acute heart failure outcomes over a 5-year period.

## Conclusion

We developed and rigorously validated the first predictive model integrating LVDDG, history of stroke, LVMI and LVEF to forecast post-ablation LV diastolic improvement in AF patients with concomitant LVDD. The model demonstrated good discrimination, acceptable calibration and meaningful clinical utility, and its performance of discrimination remained comparable in an exploratory sensitivity analysis in which LVDD was redefined according to updated diastolic-function recommendations. The resulting nomogram may support more informed patient selection for catheter ablation and help identify individuals who warrant intensive adjunctive strategies aimed at structural reverse remodelling. Future multicenter studies with systematic implementation of the full 2025 ASE diastolic-function protocol, are warranted for external validation, model refinement and assessment of its impact on clinical decision-making and outcomes.

## Data Availability

The raw data supporting the conclusions of this article will be made available by the authors, without undue reservation.

## References

[1] Naser JA Lee E Scott CG Kennedy AM Pellikka PA Lin G Prevalence and incidence of diastolic dysfunction in atrial fibrillation: clinical implications. Eur Heart J. (2023) 44(48):5049–60. 10.1093/eurheartj/ehad592 37639219 PMC13386646

[2] Seko Y Kato T Haruna T Izumi T Miyamoto S Nakane E Association between atrial fibrillation, atrial enlargement, and left ventricular geometric remodeling. Sci Rep. (2018) 8(1):6366. 10.1038/s41598-018-24875-1 29686287 PMC5913256

[3] Banerjee A Taillandier S Olesen JB Lane DA Lallemand B Lip GY Ejection fraction and outcomes in patients with atrial fibrillation and heart failure: the Loire valley atrial fibrillation project. Eur J Heart Fail. (2012) 14(3):295–301. 10.1093/eurjhf/hfs005 22294759

[4] Ikemura N Nakanishi K Spertus JA Lam CSP Kimura T Katsumata Y Left ventricular diastolic indices and their impact on outcomes in patients with recently diagnosed atrial fibrillation. J Clin Med. (2022) 11(19):5732. 10.3390/jcm11195732 36233600 PMC9571305

[5] Tzeis S Gerstenfeld EP Kalman J Saad EB Sepehri Shamloo A Andrade JG 2024 European heart rhythm association/heart rhythm society/Asia Pacific heart rhythm society/Latin American heart rhythm society expert consensus statement on catheter and surgical ablation of atrial fibrillation. Europace. (2024) 26(4):euae043. 10.1093/europace/euae043 38587017 PMC11000153

[6] Elvan A. It’s all about improvement of quality of life and reduction of disease burden in atrial fibrillation ablation. Heart. (2021) 107(16):1274–5. 10.1136/heartjnl-2021-319382 34016694

[7] Kosiuk J Breithardt OA Bode K Kornej J Arya A Piorkowski C The predictive value of echocardiographic parameters associated with left ventricular diastolic dysfunction on short- and long-term outcomes of catheter ablation of atrial fibrillation. Europace. (2014) 16(8):1168–74. 10.1093/europace/eut415 24569573

[8] Kosiuk J Buchta P Gaspar T Arya A Piorkowski C Rolf S Prevalence and predictors of worsened left ventricular diastolic dysfunction after catheter ablation of atrial fibrillation. Int J Cardiol. (2013) 168(4):3613–5. 10.1016/j.ijcard.2013.05.047 23725912

[9] Ishizuka K Hoshino T Mizuno T Takahashi S Wako S Arai S Diastolic dysfunction as a positive predictor of recurrent vascular events in patients with noncardioembolic stroke. Stroke. (2024) 55(3):595–603. 10.1161/STROKEAHA.123.042548 38328918

[10] Cavalcante JL Marek J Sheppard R Starling RC Mather PJ Alexis JD Diastolic function improvement is associated with favourable outcomes in patients with acute non-ischaemic cardiomyopathy: insights from the multicentre IMAC-2 trial. Eur Heart J Cardiovasc Imaging. (2016) 17(9):1027–35. 10.1093/ehjci/jev311 26628616 PMC5066337

[11] Masugata H Senda S Goda F Yoshihara Y Yoshikawa K Fujita N Left ventricular diastolic dysfunction in normotensive diabetic patients in various age strata. Diabetes Res Clin Pract. (2008) 79(1):91–6. 10.1016/j.diabres.2007.08.006 17919764

[12] Zheng LJ Lin X Xue YJ. Effect of cerebral ischemic strokes in different cerebral artery regions on left ventricular function. Front Cardiovasc Med. (2022) 9:782173. 10.3389/fcvm.2022.782173 35345487 PMC8957275

[13] Liang HY Hsiao YL Yeh HC Ting IW Lin CC Chiang HY Associations between myocardial diastolic dysfunction and cardiovascular mortality in chronic kidney disease: a large single-center cohort study. J Am Soc Echocardiogr. (2022) 35(4):395–407. 10.1016/j.echo.2021.12.003 34915133

[14] Wang J Li Y Guo YK Huang S Shi R Yan WF The adverse impact of coronary artery disease on left ventricle systolic and diastolic function in patients with type 2 diabetes mellitus: a 3.0 T CMR study. Cardiovasc Diabetol. (2022) 21(1):30. 10.1186/s12933-022-01467-y 35193565 PMC8864799

[15] Nagueh SF Appleton CP Gillebert TC Marino PN Oh JK Smiseth OA Recommendations for the evaluation of left ventricular diastolic function by echocardiography. J Am Soc Echocardiogr. (2009) 22(2):107–33. 10.1016/j.echo.2008.11.023 19187853

[16] Tsai JP Sung KT Su CH Lai YH Kuo JY Yun CH Diagnostic accuracy of left atrial remodelling and natriuretic peptide levels for preclinical heart failure. ESC Heart Fail. (2019) 6(4):723–32. 10.1002/ehf2.12430 30993903 PMC6676297

[17] Hindricks G Potpara T Dagres N Arbelo E Bax JJ Blomström-Lundqvist C 2020 ESC guidelines for the diagnosis and management of atrial fibrillation developed in collaboration with the European association for cardio-thoracic surgery (EACTS): the task force for the diagnosis and management of atrial fibrillation of the European Society of Cardiology (ESC) developed with the special contribution of the European heart rhythm association (EHRA) of the ESC. Eur Heart J. (2021) 42(5):507. 10.1093/eurheartj/ehaa798 32860505

[18] Nagueh SF Sanborn DY Oh JK Anderson B Billick K Derumeaux G Recommendations for the evaluation of left ventricular diastolic function by echocardiography and for heart failure with preserved ejection fraction diagnosis: an update from the American Society of Echocardiography. J Am Soc Echocardiogr. (2025) 38(7):537–69. 10.1016/j.echo.2025.03.011 40617625

[19] Cha YM Wokhlu A Asirvatham SJ Shen WK Friedman PA Munger TM Success of ablation for atrial fibrillation in isolated left ventricular diastolic dysfunction: a comparison to systolic dysfunction and normal ventricular function. Circ Arrhythm Electrophysiol. (2011) 4(5):724–32. 10.1161/CIRCEP.110.960690 21747059

[20] Liu Q Zhang Y Zhao Y You L Wu J Yin H Long-term outcome of left heart function after catheter ablation in patients with persistent atrial fibrillation combined with preserved ejection fraction heart failure. J Interv Cardiol. (2024) 2024:8332948. 10.1155/2024/8332948

[21] Kim TH Shim CY Park JH Nam CM Uhm JS Joung B Left ventricular diastolic dysfunction is associated with atrial remodeling and risk or presence of stroke in patients with paroxysmal atrial fibrillation. J Cardiol. (2016) 68(2):104–9. 10.1016/j.jjcc.2015.10.008 26603328

[22] Kucukdurmaz Z Kato R Erdem A Golcuk E Tobiume T Nagase T Catheter ablation for atrial fibrillation results in greater improvement in cardiac function in patients with low versus normal left ventricular ejection fraction. J Interv Card Electrophysiol. (2013) 37(2):179–87. 10.1007/s10840-013-9794-6 23625275

[23] Wang Gottlieb A Edner J Gottlieb-Vedi E Yacoub N Shahim A Schwieler J Long-term efficacy and safety of ablation for atrial fibrillation in patients with hypertrophic cardiomyopathy. Eur Heart J. (2024) 45(Suppl 1):626. 10.1093/eurheartj/ehae666.39338073194

[24] Einarsen E Gerdts E Waje-Andreassen U Naess H Fromm A Saeed S. Association of increased arterial stiffness with diastolic dysfunction in ischemic stroke patients: the Norwegian stroke in the young study. J Hypertens. (2020) 38(3):467–73. 10.1097/HJH.0000000000002297 31725075

[25] Donal E Grimm RA Yamada H Kim YJ Marrouche N Natale A Usefulness of Doppler assessment of pulmonary vein and left atrial appendage flow following pulmonary vein isolation of chronic atrial fibrillation in predicting recovery of left atrial function. Am J Cardiol. (2005) 95(8):941–7. 10.1016/j.amjcard.2004.12.031 15820159

[26] Mandoli GE Cameli M Pastore MC Loiacono F Righini FM D'Ascenzi F Left ventricular fibrosis as a main determinant of filling pressures and left atrial function in advanced heart failure. Eur Heart J Cardiovasc Imaging. (2024) 25(4):446–53. 10.1093/ehjci/jead340 38109280

[27] Nguyen P. Divergence between left ventricular ejection fraction and global longitudinal strain by cardiac magnetic resonance as a new predictor of myocardial fibrosis burden in hypertrophic cardiomyopathy. Cardiovasc Res. (2024) 120(Suppl 1):i141–3. 10.1093/cvr/cvae088.113

[28] Chen Z Venkat P Seyfried D Chopp M Yan T Chen J. Brain-heart interaction: cardiac complications after stroke. Circ Res. (2017) 121(4):451–68. 10.1161/CIRCRESAHA.117.311170 28775014 PMC5553569

[29] Sposato LA Hilz MJ Aspberg S Murthy SB Bahit MC Hsieh CY Post-stroke cardiovascular complications and neurogenic cardiac injury: jACC state-of-the-art review. J Am Coll Cardiol. (2020) 76(23):2768–85. 10.1016/j.jacc.2020.10.009 33272372

