# Supplementary material for: Development and validation of a predictive model for left ventricular diastolic improvement following catheter ablation in atrial fibrillation with diastolic dysfunction: a retrospective analysis
Source: Front Cardiovasc Med. 2026 Jan 12;12:1689041. doi: 10.3389/fcvm.2025.1689041 (PMC12833358; doi:10.3389/fcvm.2025.1689041)
Supplement: Supplementary file 1 [file Datasheet1.docx]

**Table Legends**

**Supplementary Table S1. Multivariate logistic regression analysis for predictors of LVDD improvement.** Abbreviations as in Table 1; *CI* = *confidence interval; OR = odds ratio.*

**Supplementary Table S2. SMDs of baseline covariates between training and validation subsets in Fold 5 of the 10-fold cross-validation.** *Continuous variables are presented as median [IQR]. Categorical variables are presented as frequency (percentage). SMDs are reported as an index of covariate balance between the two subsets; values closer to 0 indicate better balance. Abbreviations as in Table 1; SMD = standardized mean difference.*

**Figure Legends**

**Supplementary Figure S1. Weighted predictive nomogram for LVDD improvement after AF ablation.** *Points for each variable (LVDDG, LVMI, LVEF, and Stroke) are summed to obtain the total score, which corresponds to the predicted probability of improvement. Density plots along the axes show the data distribution. LVDD = left ventricular diastolic dysfunction; AF, atrial fibrillation; LVMI, left ventricular mass index; LVEF, left ventricular ejection fraction. * P < 0.05; ** P < 0.01; *** P < 0.001.*

**Supplementary Figure S2. ROC curve of the weighted predictive model.** *The weighted AUC and 95% CI (1000 bootstrap resamples) are shown. ROC* = *receiver operating characteristic; AUC* = *area under the curve; CI* = *confidence interval.*

**Figures**

**Supplementary Figure S1**

**
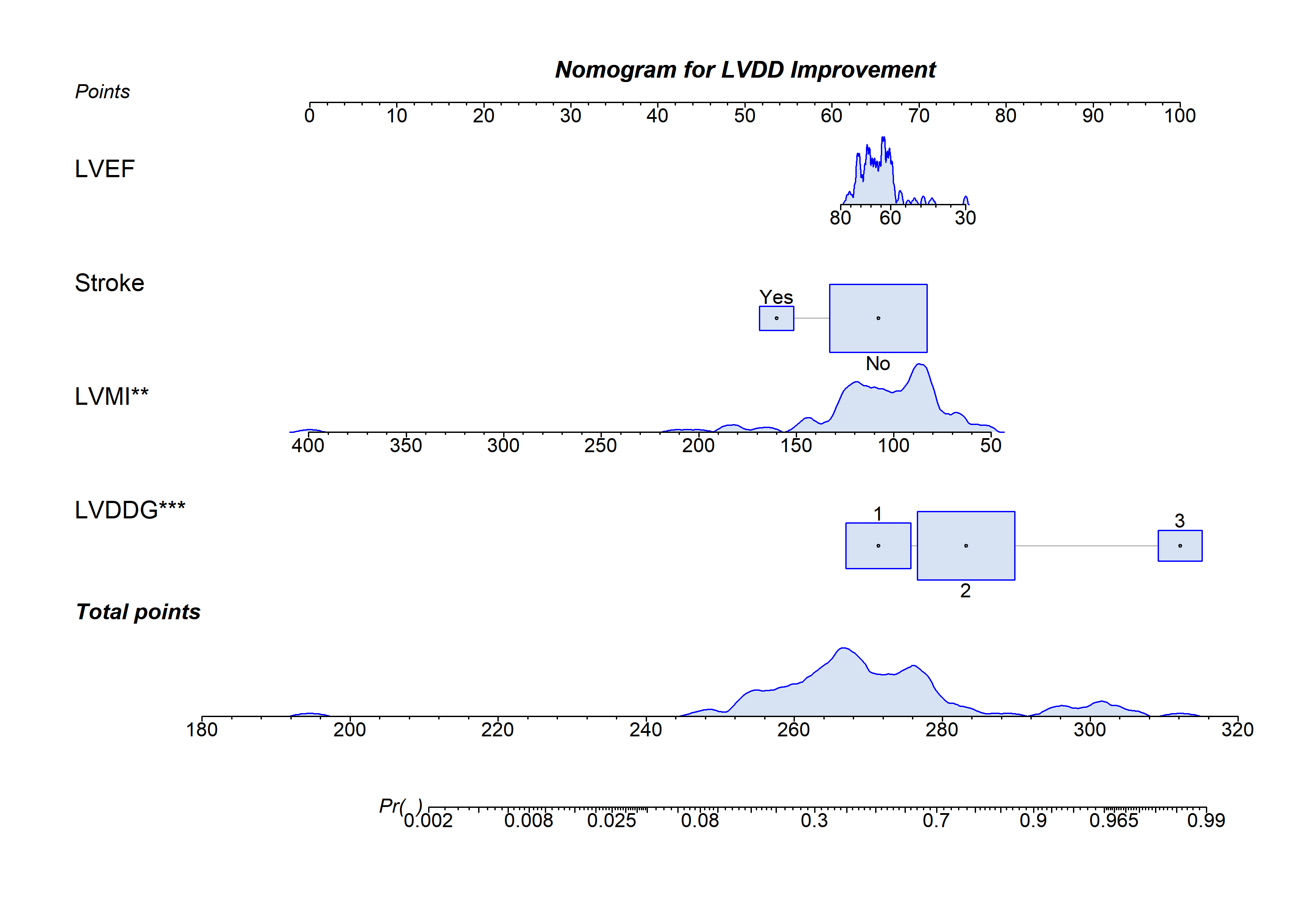
**

**Supplementary Figure S2.**

**
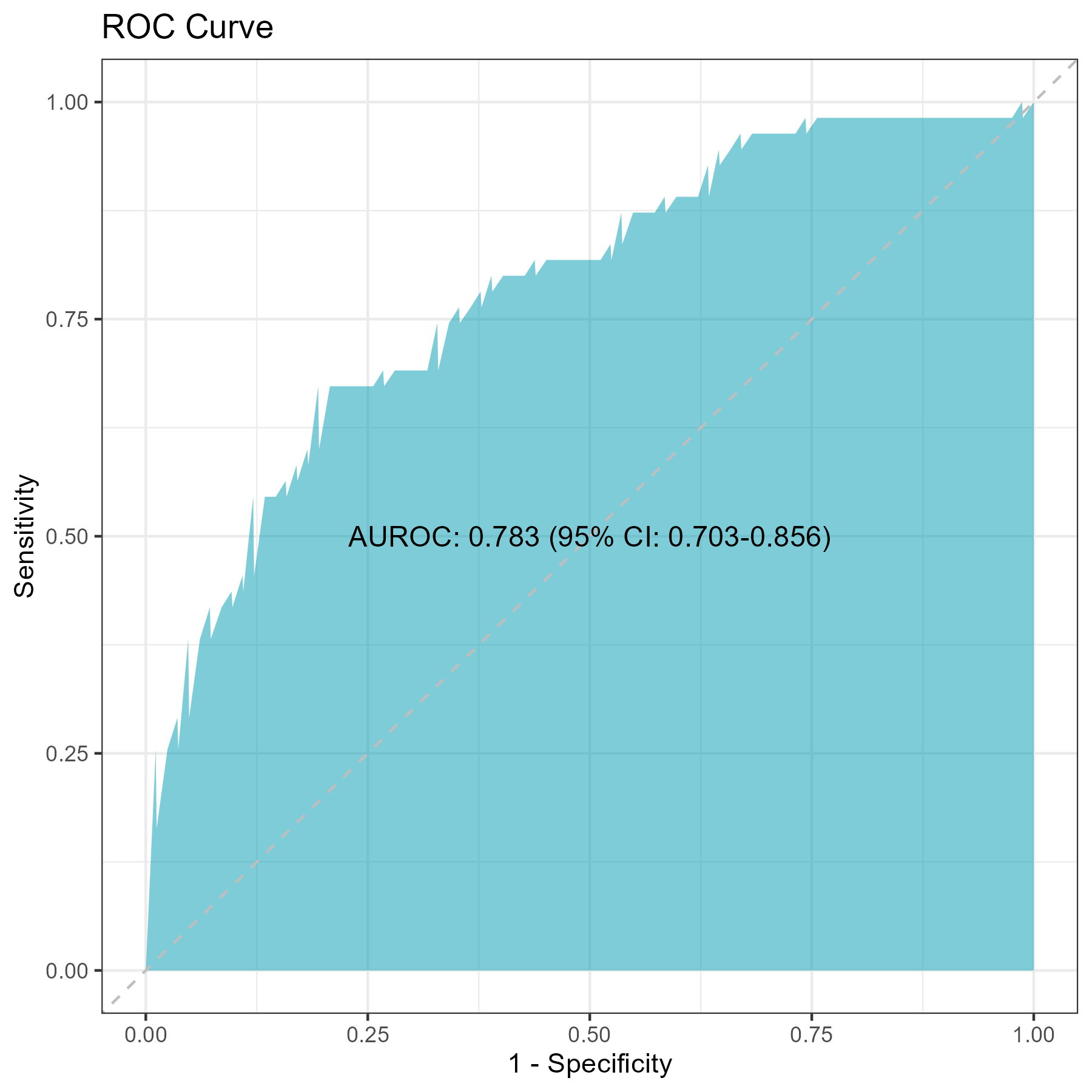
**

**Tables**

**Supplementary Table S1.**

| Variable | OR | 95% CI | p-value |
| --- | --- | --- | --- |
| LVDDG (level 2 vs 1) | 2.82 | 1.12 - 7.08 | 0.028 |
| LVDDG (level 3 vs 1) | 35.40 | 5.20 - 241.10 | < 0.001 |
| History of Stroke | 0.30 | 0.08 - 1.20 | 0.088 |
| LVMI | 0.98 | 0.96 - 0.99 | 0.003 |
| LVEF | 0.97 | 0.92 - 1.02 | 0.246 |

**Supplementary Table S2.**

| Feature | Training Set (n=127) | Validation Set (n=14) | SMD |
| --- | --- | --- | --- |
| Age (years), median [IQR] | 68.00 [62.00 , 75.00] | 61.00 [58.50 , 67.75] | 0.424 |
| LVMI (g/m²), median [IQR] | 103.45 [85.84, 122.35] | 100.41[89.03 , 112.37] | 0.205 |
| RWT, median [IQR] | 0.38 [0.35, 0.42] | 0.38 [0.35, 0.40] | 0.270 |
| LVEF (%), median [IQR] | 66.00 [61.00, 70.00] | 68.50 [63.25, 69.75] | 0.018 |
| eGFR(mL/min/1.73 m²), median [IQR] | 79.64 [65.28, 90.50] | 84.06 (68.67, 93.75) | -0.157 |
| Gender (Male), n(%) | 64 (50.4) | 6 (42.9) | -0.151 |
| AF type(Paroxysmal), n(%) | 109 (85.8) | 13 (92.9) | 0.206 |
| LVDDG |  |  | 0.224 |
| LVDDG1, n(%) | 58 (45.7) | 7 (50.0) | - |
| LVDDG2, n(%) | 50 (39.4) | 6 (42.9) | - |
| LVDDG3, n(%) | 19 (15.0) | 1 (7.1) | - |
| Mitral Regurgitation, n(%) | 37 (29.1) | 1 (7.1) | 0.496 |
| Tricuspid Regurgitation, n(%) | 48 (37.8) | 1 (7.1) | 0.664 |
| Smoking, n(%) | 25 (19.7) | 2 (14.3) | 0.137 |
| Drinking, n(%) | 19 (15.0) | 3 (21.4) | 0.178 |
| Redo Ablation, n(%) | 16 (12.6) | 3 (21.4) | -0.259 |
| Hypertension, n(%) | 69 (54.3) | 7 (50.0) | 0.087 |
| Diabetes Mellitus, n(%) | 22 (17.3) | 2 (14.3) | 0.081 |
| History of Stroke, n(%) | 15 (11.8) | 1 (7.1) | 0.147 |
| Coronary Artery Disease, n(%) | 12 (9.4) | 2 (14.3) | -0.162 |
| Procedure Characteristics |  |  |  |
| Ablation Energy(cryoablation), n(%) | 10(7.9) | 1(7.1) | 0.027 |
| LAAO, n(%) | 27(21.3) | 2(14.3) | 0.173 |
| Additional Ablation, n(%) | 17(13.4) | 3(21.4) | -0.231 |
